# Review of Recent Development of MEMS Speakers

**DOI:** 10.3390/mi12101257

**Published:** 2021-10-16

**Authors:** Haoran Wang, Yifei Ma, Qincheng Zheng, Ke Cao, Yao Lu, Huikai Xie

**Affiliations:** 1 Department of Electrical and Computer Engineering, University of Florida, Gainesville, FL 32611, USA; wanghaoran@ufl.edu; 2 School of Information and Electronics, Beijing Institute of Technology, Beijing 100081, China; 3120200697@bit.edu.cn (Y.M.); 1120171582@bit.edu.cn (Q.Z.); 3220210564@bit.edu.cn (K.C.); y.lu@bit.edu.cn (Y.L.); 3 BIT Chongqing Center for Microelectronics and Microsystems, Chongqing 400030, China

**Keywords:** microelectromechanical systems, MEMS, loudspeakers, microspeakers

## Abstract

Facilitated by microelectromechanical systems (MEMS) technology, MEMS speakers or microspeakers have been rapidly developed during the past decade to meet the requirements of the flourishing audio market. With advantages of a small footprint, low cost, and easy assembly, MEMS speakers are drawing extensive attention for potential applications in hearing instruments, portable electronics, and the Internet of Things (IoT). MEMS speakers based on different transduction mechanisms, including piezoelectric, electrodynamic, electrostatic, and thermoacoustic actuation, have been developed and significant progresses have been made in commercialization in the last few years. In this article, the principle and modeling of each MEMS speaker type is briefly introduced first. Then, the development of MEMS speakers is reviewed with key specifications of state-of-the-art MEMS speakers summarized. The advantages and challenges of all four types of MEMS speakers are compared and discussed. New approaches to improve sound pressure levels (SPLs) of MEMS speakers are also proposed. Finally, the remaining challenges and outlook of MEMS speakers are given.

## 1. Introduction

With the rapid advancement of consumer electronics, the worldwide audio market has been seeing a growing trend towards smaller devices with lower power consumption and better performance in the last decade. Speakers, as one of the core components in mobile electronic devices such as laptops, smartphones, wireless earbuds, and human-machine interfaces, are highly demanded to be smaller, lighter, and more power efficient. Currently, speakers in those mobile electronic devices are dominated by conventional speakers with bulky moving coils, which are still challenging to be batch fabricated since voice coils and permanent magnets must be assembled [1]. The miniaturization of these conventional speakers also has a negative impact on the sound quality and reaches some limits due to the employed materials and the fabrication approaches [2]. For example, the plastic or polymer diaphragms of conventional speakers are too soft to be used as high-quality radiator surfaces [3]. The simplification of the mechanical suspensions and electromagnetic parts in the miniaturization would lead to reduced bandwidths and increased nonlinearities, thus deteriorating the sound quality [2,4]. It is also difficult for conventional manufacturing technologies to achieve high dimensional precision and good reproducibility in the miniaturization of speakers.

By contrast, microelectromechanical systems (MEMS) speakers, or microspeakers, have been drawing more and more attention due to their inherent advantages, e.g., small form factors, low power consumption, batch fabrication, and potential on-chip integration with electronic circuits. Many researchers have developed MEMS speakers based on various transduction mechanisms and achieved promising results, including electrodynamic MEMS speakers [5,6,7], electrostatic MEMS speakers [8,9], piezoelectric MEMS speakers [10,11,12], and thermoacoustic MEMS speakers [13,14]. Various materials and fabrication approaches have also been explored for developing MEMS speakers [15,16,17]. The performances of MEMS speakers have been evaluated and compared with conventional speakers in terms of several key specifications, such as device footprint, output sound pressure level (SPL), power consumption, bandwidth, and total harmonic distortion (THD) [2,9,10,17]. Among them, the SPL and bandwidth are two widely used parameters to evaluate the acoustic performance of MEMS speakers. THD, defined as the sum of all power radiated in frequencies other than the fundamental frequency relative to the total emitted sound power, is an important parameter to evaluate the sound quality of MEMS speakers [9].

To date, MEMS speakers have been developed mainly for in-ear applications (e.g., hearing aids) and headphones [15,18]. It is challenging for MEMS speakers and conventional electrodynamic microspeakers as well to achieve both high SPL output and flat audio frequency response due to the vibration mode complexity of the diaphragm and the limited space for actuation. Thus, the actuation method, structure, and electrode pattern design of the diaphragm as well as the enclosure design are crucial to the overall response and performance of a MEMS speaker. Both finite element analysis (FEA) and lumped element modelling (LEM) are typically employed to study the effects of various design parameters and to optimize the overall performance of MEMS speakers [19,20]. Several approaches in terms of material selection [12], special structural design [21,22], and electrode configuration [23,24] have also been demonstrated to achieve the better acoustic performance of MEMS speakers. Extensive research efforts have been devoted to developing better MEMS speakers with promising results demonstrated, which is evidenced by a large amount of literature produced.

With so many research efforts paid to the development of MEMS speakers, significant progress has been made in their commercialization. For example, piezoelectric MEMS speakers developed by Usound have reached the market. With a chip size of 6.7 mm × 4.7 mm × 1.58 mm, the developed piezoelectric MEMS speaker can generate a high SPL of around 116 dB in an acoustic coupler, under a driving voltage of 15 V [25]. The TDK Corporation has developed a series of piezoelectric speakers called PiezoListen. With a thickness of as small as 0.49 mm and footprints ranging from 20 mm × 10 mm to 66 mm × 30 mm, the developed speakers can be installed on almost any kinds of displays or surfaces to generate sound over a wide frequency range from 400 Hz to 20 kHz [26]. In addition, Audio Pixels has successfully implemented a digital sound reconstruction (DSR) technique in a commercially feasible manner and developed MEMS speaker arrays to generate high quality sounds [27]. Furthermore, by using moving beams with electrostatic actuation to generate sound inside silicon chips, Arioso Systems has developed MEMS speakers with high-fidelity sound and CMOS-compatible process for in-ear applications [28].

In order to better leverage the existing achievements, it is necessary to sort out the recent development of MEMS speakers, understand the barriers, compare different types of MEMS speakers, and point out the future perspectives with respect to these challenges. Thus, the main purpose of this article is to provide a state-of-the-art review of MEMS speakers and a future outlook as well.

This review article is organized as follows. In Section 2, we introduce the theories and modeling of MEMS speakers, including device concepts, LEMs, and several transduction mechanisms. In Section 3, we review different types of MEMS speakers, including their fabrication technologies, characterization results, and approaches to improve the SPLs of MEMS speakers with regard to structures, materials, and actuation methods. The focus is on the piezoelectric MEMS speaker. In Section 4, we compare and discuss the performances of different MEMS speakers. In Section 5, we summarize the review and discuss future perspectives of MEMS speakers.

## 2. Theory and Modeling of MEMS Speakers

### 2.1. Basic Structure

**Figure 1:**
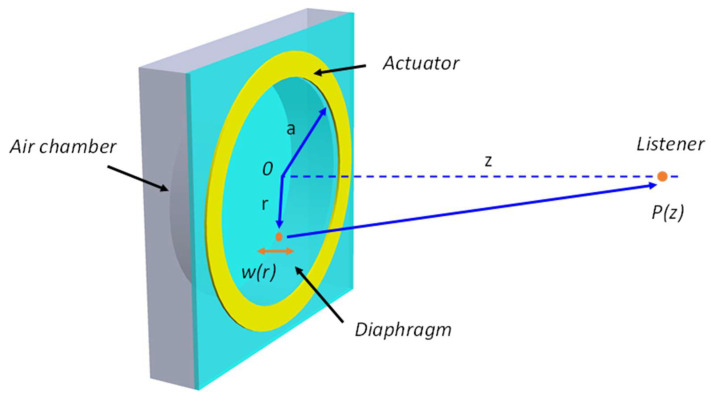
Schematic of a MEMS speaker with a piston-move diaphragm and the geometries for sound pressure calculation.

In general, the main structure of a MEMS speaker consists of an acoustic diaphragm, an actuation mechanism, and an air chamber. When an AC voltage is applied to drive the MEMS speaker, a bending moment will be generated by the actuation mechanism, forcing the diaphragm to vibrate and thus generating a sound pressure output. Considering a circular vibrating diaphragm, as shown in Figure 1, the pressure amplitude can be calculated based on the Helmholtz equation and the Rayleigh integral and is readily given by [29]:

(1) $$P\left(z\right)=\rho {\left(2\pi f\right)}^{2}\int_{0}^{a}\frac{w\left(r\right)}{\sqrt{z^{2}+r^{2}}}rdr$$

where ρ is the air density, f is the vibration frequency, *a* is the radius of the diaphragm, w(r) is the vibration amplitude at the radial distance of r, and z is the distance from the diaphragm to the listener.

When the vibration of the acoustic diaphragm is simplified as a piston on an infinite baffle, the effective sound pressure output $P_{e}\left(z\right)$ and the sound pressure level (SPL) in decibels (dB) can be further simplified as:

(2) $$P_{e}\left(z\right)=\frac{P\left(z\right)}{\sqrt{2}}=\frac{\sqrt{2}\pi \rho Swf^{2}}{z}$$

(3) $$SPL=20\;\mathrm{lg}\left(\frac{P_{e}\left(z\right)}{P_{ref}}\right)$$

where S and w are the surface area and vibration amplitude of the diaphragm, respectively. The reference effective sound pressure value $P_{ref}$ is 20 μPa [30]. Typically, the SPLs of MEMS speakers are measured by microphones placed at 1 cm away from the MEMS speakers in open air. For MEMS speakers specifically developed for in-ear or hearing-aid applications, their SPLs are measured in a 2cc coupler (a coupler with a volume of 2 cm^3^ that conforms to the ANSI S3.7 and IEC 60318-5 standards) [31].

**Figure 2:**
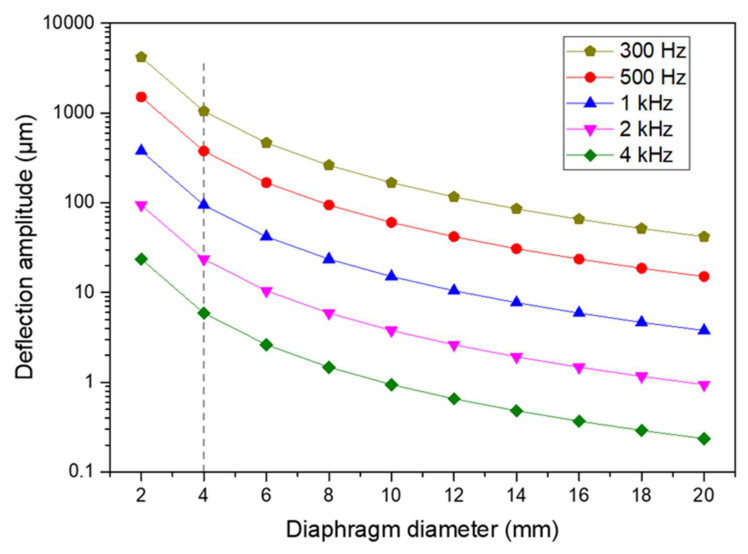
Required deflection amplitudes for different diaphragm diameters to achieve a 90 dB SPL at 1 cm at frequencies of 300 Hz, 500 Hz, 1 kHz, 2 kHz, and 4 kHz.

The acoustic diaphragm is important in MEMS speaker designs. According to Equation (2), the sound pressure output generated by the acoustic diaphragm is proportional to its surface area and vibration amplitude, and the square of the working frequency. Thus, generating high sound pressure output at lower frequencies is more challenging, which requires larger deflections under the same diaphragm size constraint, as indicated in Figure 2. Figure 2 plots the required deflection amplitudes for circular diaphragms with different frequencies and different diameters to achieve a 90 dB SPL at 1 cm. This plot shows the decreasing trend of the required deflection amplitudes with the increasing frequencies and the diaphragm sizes and gives a general indication of the design values. As can be seen, for a circular diaphragm with a diameter of 4 mm, achieving a 90 dB SPL at 1 cm requires a diaphragm deflection of 5.9 μm, 94.4 μm, and 1.05 mm at frequencies of 4 kHz, 1 kHz, and 300 Hz, respectively.

MEMS speakers are usually designed to work in a frequency range from 20 Hz to 20 kHz, which is consistent with the hearing range of humans. Since the frequencies of audible sounds for humans in daily life typically varies from 100 Hz to 10 kHz, including speeches in low frequencies (300 Hz–3.4 kHz) and musical harmonics in high frequencies (>6 kHz) [32], MEMS speakers are normally evaluated in both of these low-frequency and high-frequency bands. In general, piezoelectric, electrodynamic, and electrostatic actuation are the three most commonly used approaches to excite acoustic diaphragms. Details of these transduction mechanisms will be introduced in Section 2.2.

In addition to the deflection, resonant frequency is another important design parameter of acoustic diaphragms. Most of MEMS speakers presented in literatures are developed based on deformable diaphragms with edges clamped on the substrate. Their fundamental resonant frequencies are dependent on the dimensions and material properties of the diaphragms. For a circular clamped vibrating diaphragm, the fundamental resonant frequency $f_{0}$ is given by [33]:

(4) $$f_{0}=0.47\frac{t}{a^{2}}\sqrt{\frac{E}{\rho_{m}\left(1-v^{2}\right)}}$$

where t, a, E, $\rho_{m}$, and v are the thickness, radius, effective Young’s modulus, mass density, and Poisson’s ratio of the circular diaphragm, respectively.

The fundamental vibration mode of the clamped diaphragm is the so-called drum mode, whose deflection profile peaks at the center of the diaphragm and decreases from the center to the edge. When designing the fundamental resonant frequency, there are two considerations. On one hand, to achieve high SPL at low frequencies and thus improve the acoustic performance over a wide frequency range, the fundamental drum mode frequencies are typically designed at around 2 kHz to 3 kHz [16,34,35]. On the other hand, from the acoustic point of view, the drum mode vibration with the deformed emissive surface and higher harmonics stimulation due to nonlinearities will distort the acoustic wavefront, therefore causing sound distortions and deteriorating the sound quality [2]. Thus, some special diaphragm designs other than edge clamped diaphragms have been developed, such as rigid diaphragms with radial rib structures supported by suspension beams [2] and circular diaphragms supported by four flexible dual-curve actuators [21], in which piston mode vibrations at low frequencies can be employed to generate the sound while the drum mode vibrations can be shifted to high frequencies to avoid the sound distortion of MEMS speakers.

### 2.2. Transduction Mechanisms

**Figure 3:**
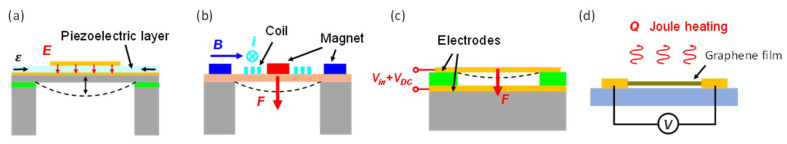
Schematic of MEMS speakers based on (**a**) piezoelectric, (**b**) electrodynamic, (**c**) electrostatic, and (**d**) thermoacoustic transduction mechanisms.

MEMS speakers have been developed based on various transduction mechanisms, including the piezoelectric transduction [36], electrodynamic transduction [37], electrostatic transduction [38], and thermoacoustic transduction [13]. Figure 3 shows the schematics of MEMS speakers with different transduction mechanisms. Among them, MEMS speakers developed based on the first three types of transduction mechanisms rely on the mechanical vibration of the acoustic diaphragm to generate the sound. By contrast, thermoacoustic MEMS speakers produce the sound by the periodic contraction and expansion of the medium around the diaphragm due to the heat exchange between the diaphragm and the surrounding medium.

As shown in Figure 3a, piezoelectric MEMS speakers work on the flexural vibration of the piezoelectric diaphragm. When an AC voltage is applied across the piezoelectric film sandwiched by two metal electrodes, an in-plane strain will be generated based on the converse piezoelectric effect, thus causing the out-of-plane vibration of the diaphragm. The relation between the in-plane strain ε and the applied electric field E can be expressed by [21]:

(5) $$\varepsilon =d_{31}E$$

where $d_{31}$ is the piezoelectric constant of the employed piezoelectric film.

For electrodynamic MEMS speakers, the acoustic diaphragm is actuated by electromagnetic (Lorentz) force. As shown in Figure 3b, when the current flows through coils, Lorentz force will be generated due to the interaction between the external magnetic field and the electric current, thus bending the acoustic diaphragm. For a planar concentric coil with N turns carrying an electric current I, the Lorentz force $F_{Lorentz}$ generated by a magnetic field with a flux density B can be expressed as [2]:

(6) $$F_{Lorentz}=I\int_{0}^{l}\vec{B}d\vec{l}=\overset{N}{\underset{i=1}{\sum }}2\pi IR_{i}B_{i}$$

where l is the length of the coil, $R_{i}$ is the radius of the *i*th turn, and $B_{i}$ is the radial component of the magnetic flux density on the coil plane corresponding to the *i*th turn.

Electrostatic MEMS speakers are driven by the electrostatic force between two conductive plates. As shown in Figure 3c, the acoustic diaphragm is suspended over the substrate by a small gap d. Considering this structure as a parallel-plate capacitor with flat and rigid electrodes for simplification, the electrostatic force exerted on the diaphragm under an AC driving voltage $V_{in}$ and a DC bias $V_{DC}$ is given by [39]:

(7) $$F_{E}=\frac{1}{2}\epsilon A{\left(\frac{V_{in}+V_{DC}}{d}\right)}^{2}$$

where ϵ is the electric permittivity of air and A is the area of the diaphragm. Advanced models considering the bending of the plate and pull-in limitations are presented in [40,41].

Different from the mechanical vibration sound generators described above, thermoacoustic MEMS speakers emit sound by the thermoacoustic effect, which converts the Joule heat into sound. As shown in Figure 3d, when an AC current is applied to a conductive film, the film will be heated and exchange the thermal energy with the surrounding air, causing the periodic contraction and expansion of the air, thus generating sounds. The root-mean-square sound pressure amplitude produced by a thermoacoustic thin film speakers can be derived as [42]:

(8) $$p_{rms}=\frac{\sqrt{\alpha }\rho_{0}}{2\sqrt{\pi }T_{0}}\cdot \frac{1}{r}\cdot P_{in}\cdot \frac{\sqrt{f}}{C_{s}}\cdot M$$

where $\rho_{0}$, α, and $T_{0}$ are the mass density, thermal diffusivity, and temperature of the ambient gas, respectively, r is the distance between the thin film conductor and the listener, $P_{in}$ is the input power, f is the frequency of the sound, $C_{s}$ is the heat capacity per unit area of the thin film conductor, and M is a frequency-related factor.

### 2.3. Modeling

The acoustic performance of MEMS speakers is dependent on many design parameters, including material properties, device structures, and acoustic enclosure designs. Lumped element modeling (LEM) and finite element analysis (FEA) can be used to effectively predict the acoustic performance of MEMS speakers and optimize the designs. For example, Neumann Jr. et al. presented CMOS-MEMS diaphragms for acoustic actuation based on electrostatic force, and developed a simplified acoustic model to investigate the effects of the dimensional parameters of the diaphragms [43]. Huang et al. studied the sound pressure response of miniaturized moving-coil loudspeakers using an equivalent circuit method (ECM), which can simulate the electrical, mechanical, and acoustical responses and optimize the device designs [44]. These methods are also called electro-mechano-acoustical modeling, or lumped element modeling (LEM), which can be applied to study the effects of different acoustic enclosures and model the performances of MEMS speakers based on different transduction mechanisms [16,45]. LEM is a simple and efficient tool for designing and analyzing multiphysics systems as well as for predicting their responses. In this method, the representation of spatially distributed physical systems is simplified by using a set of lumped elements when the length scale of the device is much smaller than the wavelength of the governing physical phenomenon. Since the acoustic wavelengths (34.3–343 mm for 1–10 kHz) for MEMS speakers are much greater than their sizes (1–10 mm), LEM is applicable.

**Figure 4:**
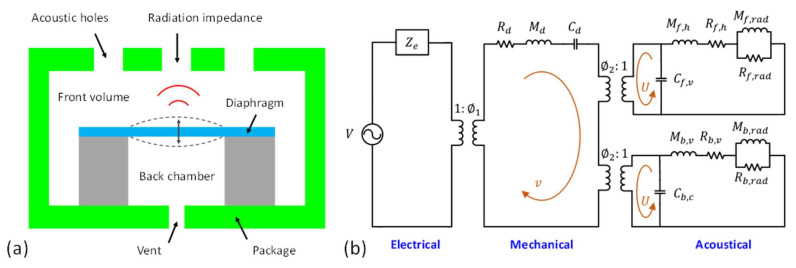
Lumped element model (LEM) of a packaged MEMS speaker: (**a**) illustration of the structures and (**b**) the equivalent circuit in multiple domains.

Typically, MEMS speakers are packaged in enclosures with a front cover, a back chamber, and vent holes. Figure 4a illustrates a simplified structure of a MEMS speaker in a package. The LEM of this device is shown in Figure 4b, representing a multiphysics system consisting of electrical, mechanical, and acoustical energy domains. In the electrical domain, the effort and flow are voltage (in V) and current (in A), respectively. The electrical and mechanical domains are coupled by a transformer or a gyrator that models the transduction mechanism of the MEMS speaker. In the mechanical domain, the effort represents the force (in N) that actuates the vibrating diaphragm while the flow represents the velocity of the diaphragm (in m/s). The acoustical domain is coupled to the mechanical domain by the effective area of the diaphragm. Thus, the effort and flow in the acoustical domain correspond to the pressure (in Pa) and volume velocity (in m^3^/s), respectively. The lumped elements sharing the same effort are connected in parallel, while those sharing the same flow are connected in series.

In the electrical domain, the electrical input impedance of the MEMS speaker is modeled as $Z_{e}$, which can be resistance and inductance from the wires and coils for electrodynamic MEMS speakers, or capacitance and resistance for electrostatic MEMS speakers and piezoelectric MEMS speakers. The electrical domain is coupled to the mechanical domain by a transformer (or a gyrator), representing the energy transformation from the electromagnetic force, electrostatic force, or the piezoelectric force.

In the mechanical domain, the vibrating diaphragm is modeled as a mass-spring-damper system, governed by the following equation:

(9) $$M_{d}\frac{d^{2}w}{dt^{2}}+R_{d}\frac{dw}{dt}+\frac{w}{C_{d}}=F_{t}$$

where w is the vibration amplitude of the diaphragm, $F_{t}$ is the total force applied on the diaphragm, and $M_{d}$, $C_{d}$, and $R_{d}$ are the equivalent mass, compliance, and damping of the diaphragm, respectively. The mechanical and acoustical domains are coupled with the effective area of the diaphragm, which converts the actuation force to the acoustic pressure. Two separate transformers are used to account for the front and the back sides of the diaphragm [44].

In the acoustical domain, the air in an acoustic chamber with a volume $V_{a}$ can be modeled as an acoustic compliance $C_{a}$ that is readily given by [44]:

(10) $$C_{a}=\frac{V_{a}}{\rho_{a}c^{2}}$$

where $\rho_{a}$ and c are the air density and the sound speed, respectively. Therefore, the front volume and the back chamber can be modeled as acoustic compliances $C_{f,v}$ and $C_{b,c}$, respectively. The air flow inside narrow spaces can be modeled as acoustic resistances and masses, such as $R_{f,h}$ and $M_{f,h}$ of the acoustic holes in the front side and $R_{b,v}$ and $M_{b,v}$ of the backside vent. The acoustic radiation impedance of the diaphragm is also approximated as the acoustic resistance $R_{f,rad}$ ($R_{b,rad}$) and mass $M_{f,rad}$ ($M_{b,rad}$). Details of the calculation of these lumped elements are described in [44].

By solving the equivalent circuit in the LEM, the volume velocity U generated by the MEMS speaker in the acoustical domain can be obtained. Thus, by assuming the MEMS speaker as a point source at a far-field distance r (much larger than the Rayleigh distance), the sound pressure output P can be calculated as [45]:

(11) $$P=j\frac{k\rho_{a}c}{2\pi r}\cdot U\cdot e^{j\left(\omega t-kr\right)}$$

where k and ω are the wave number and angular frequency of the acoustic wave, respectively. Here, it is worthy of note that the MEMS speaker is considered as a monopole mounted on a baffle plate for far-field calculation. Since the plate restricts the acoustic radiation only to the forward hemisphere, the pressure is twice that of a free radiation without a baffle plate [46].

The LEM has been widely applied to predict the dynamic responses of MEMS speakers, especially at low-frequency regions or in the neighborhood of the fundamental resonant frequency due to its simplicity [16,47]. However, the LEM is not sufficient to model higher order resonant modes and incapable to well predict the high frequency responses of MEMS speakers. Therefore, LEM is often used together with FEA to calculate the dynamic responses [5,44,48], analyze the enclosure designs [45,49], and optimize the diaphragm structural designs.

## 3. Development of MEMS Speakers

The study of MEMS speakers started in the late 1990s. Since then, significant progress has been made to develop MEMS speakers based on different transduction mechanisms, especially on piezoelectric, electrodynamic, and electrostatic transduction. To achieve a small size, high output sound pressure, and flat frequency response, various materials, structure designs, and fabrication techniques have been employed. In this section, the development of MEMS speakers will be reviewed based on their transduction mechanisms.

### 3.1. Piezoelectric MEMS Speakers

#### 3.1.1. Design and Fabrication of Piezoelectric MEMS Speakers

**Figure 5:**
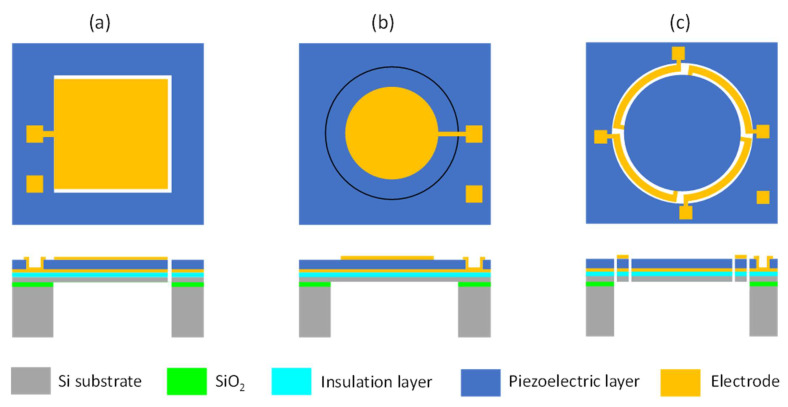
Schematics of typical structures of piezoelectric MEMS speakers in top view (**top**) and cross-sectional view (**bottom**). (**a**) Beam-like piezoelectric actuator. (**b**) Fully clamped diaphragm with piezoelectric layer embedded. (**c**) Partially clamped diaphragm surrounded by piezoelectric actuators.

Piezoelectric actuation, with the advantages of small driving voltage and large actuation force, has been widely used in many MEMS devices, including ink-jet printer heads [50], MEMS scanning mirrors [51], ultrasonic motors [52], RF resonators [53], and acoustic generators [54]. Among them, piezoelectric MEMS speakers are important applications and are attracting more and more interest. Piezoelectric MEMS speakers based on different piezoelectric materials, such as zinc oxide (ZnO), aluminum nitride (AlN), and lead zirconate titanate (PZT), have been presented for hearing aid or earphone applications [35,55,56]. Piezoelectric MEMS speakers mainly consist of a piezoelectric vibration diaphragm and an acoustic cavity. Typical vibration diaphragms can be designed as beam-like piezoelectric actuators [57] (Figure 5a), fully clamped diaphragms with piezoelectric layers embedded [12] (Figure 5b), or partially clamped diaphragms surrounded by piezoelectric actuators [21] (Figure 5c). Various piezoelectric MEMS speakers based on different designs have been demonstrated [12,58].

**Figure 6:**
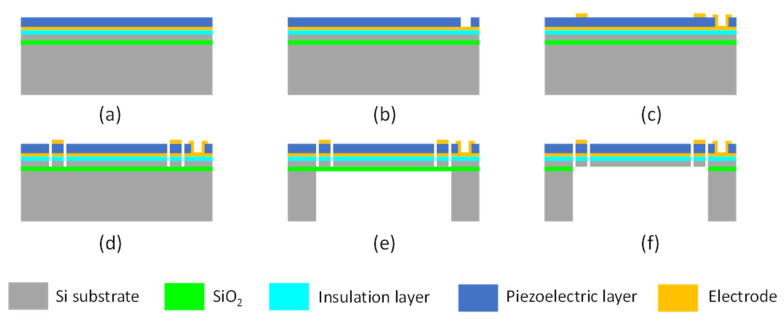
Typical fabrication process flow of a piezoelectric MEMS speaker. (**a**) Deposit insulation, electrode, and piezoelectric layers. (**b**) Pattern the piezoelectric layer. (**c**) Deposit and pattern the top electrode layer. (**d**) Reactive ion etching to define the diaphragm and piezoelectric actuators. (**e**) Etch the acoustic cavity. (**f**) Release the moveable structures.

The fabrication process of piezoelectric MEMS speakers with various structures can be different, depending on whether the diaphragm needs to be released from both sides (Figure 5a,c) or the backside only (Figure 5b), but their general steps are similar. Here, an example for the design of MEMS speakers with a partially clamped diaphragm (Figure 5c) is presented to illustrate the typical fabrication process. As shown in Figure 6, firstly, an insulation layer (Si_x_N_y_ or SiO_2_), a bottom electrode layer, and a piezoelectric layer are deposited in sequence on a silicon-on-insulator (SOI) substrate (Figure 6a). After that, the piezoelectric layer is patterned by wet etching or reactive ion etching (RIE) to expose the bottom electrode [59,60] (Figure 6b). Next, a top electrode is deposited and patterned (Figure 6c). After that, RIE is used to define a diaphragm and a set of piezoelectric actuators on the front side (Figure 6d). Subsequently, the acoustic cavity is defined on the backside with a two-sided photolithography and formed by the deep reactive ion etching (DRIE) of silicon or wet etching with KOH (Figure 6e). The buried oxide layer is used as the etch stop and finally removed by RIE or vapor hydrofluoric acid to release the moveable structures (Figure 6f). For the fabrication of fully clamped diaphragms in Figure 5b, the process step shown in Figure 6d can be skipped.

In the design and fabrication of piezoelectric MEMS speakers, the material of the piezoelectric layer is important as it will affect the selection of the fabrication method and the performance of the fabricated devices. Next, the piezoelectric materials for making MEMS speakers will be discussed.

#### 3.1.2. Piezoelectric Materials

Lead zirconate titanate (PZT) ceramics, single-crystal lithium niobate (LiNbO_3_), and single-crystal lead magnesium niobate-lead titanate (PMN-PT) are widely used bulk piezoelectric materials with high piezoelectric coefficients and electromechanical coupling factors for piezoelectric transducers [61]. However, how to thin down these materials remains an issue in fabricating piezoelectric MEMS devices. With the advancement of thin film deposition technologies, piezoelectric thin films including ZnO, AlN, and PZT can be fabricated by sputtering or sol-gel methods, which have been applied to fabricate piezoelectric MEMS devices, such as microspeakers [62,63]. Among these materials, ZnO is one of the most commonly used for making piezoelectric thin film devices such as film bulk acoustic wave resonators (FBAR), surface acoustic wave (SAW) resonators, piezoelectric micromachined ultrasonic transducers (pMUTs), and microspeakers in early years. ZnO-based piezoelectric MEMS speakers have been developed as early as in 1996, when Lee et al. fabricated a piezoelectric cantilever transducer that worked both as a microphone and a microspeaker [58]. In their system, the 2000 × 2000 × 4.5 μm^3^ piezoelectric cantilever was fabricated based on a 0.5 μm-thick ZnO layer with the magnetron sputtering method. In 2003, Ko et al. presented a piezoelectric microspeaker based on a clamped 3000 × 3000 × 3 μm^3^ diaphragm. This micromachined transducer also has a thin ZnO film as the piezoelectric layer, which is deposited on a membrane of low-stress silicon nitride of 1.5 μm [64].

Another type of piezoelectric material, AlN, has also been well studied and characterized in the past few decades. A thin film of AlN is normally deposited by the reactive magnetron sputtering method. Sputtered AlN thin films have better chemical and thermal stability than ZnO. The lower conductivity of AlN compared to ZnO also results in lower power loss [65]. With these advantages, AlN has also been a good candidate for fabricating the piezoelectric layer of MEMS speakers. In 2007, Seo et al. presented piezoelectric microspeakers with circular-type and cross-type electrode configurations based on a 0.5-μm-thick AlN film [36]. With a diaphragm size of 4 × 4 mm^2^, the AlN-based microspeakers achieved good acoustic performance with a high sound pressure level (SPL).

However, it is challenging to sputter ZnO and AlN with controlled properties. Their morphology and crystalline quality will highly affect the piezoelectric constants of materials. In a fabrication process, the sputtering rate and residual stress are dependent on the sputtering condition and film thicknesses [66,67]. Sputtering with heated substrates (above 300 ℃) have been reported with large residual stresses [35,68], which will wrinkle the diaphragm of fabricated piezoelectric MEMS speakers and affect the sound pressure output. It is possible to deal with such residual stress problem by adding a stress compensation layer or fabricating dome-shaped diaphragms to reduce the effect of the residual stress. For example, in 2000, Han et al. reported dome-shaped piezoelectric MEMS speakers built on 1.5-μm-thick Parylene diaphragms, which can easily release the residual stress through volumetric shrinkage or expansion [69]. In 2009, Yi et al. reported piezoelectric AlN MEMS speakers with improved performance by controlling the residual stress of the compressively stressed diaphragm using Si_x_N_y_ films [35]. The results revealed that the SPLs of the piezoelectric AlN microspeakers were increased by more than 10 dB when the residual stresses became more compressive, especially at the low frequency region.

Other limitations of sputtering ZnO and AlN thin films include low deposition rates (tens of nm/min), small film thicknesses, and small piezoelectric constants [67,70]. The lower value of piezoelectric constants will directly limit the vibration amplitude of a piezoelectric diaphragm and lead to poor acoustic performance. By contrast, PZT thin films have greater piezoelectric constants and are favorable for the applications of piezoelectric actuation. The sputtering and sol-gel methods have also been employed to deposit PZT thin films with typical thicknesses of 0.5–2 μm, which can be applied to a wide range of applications [63]. For example, in 2009, Cho et al. fabricated a piezoelectric MEMS speaker based on a sol-gel PZT thin film with a thickness of 700 nm [11]. The fabricated MEMS speaker had a circular diaphragm with a diameter of 2 mm, which achieved SPLs of 79 dB at 1 kHz, 87 dB at 5 kHz, and 90 dB at 10 kHz under a driving voltage of 13 V. However, sputtered and sol-gel PZT films also suffer from residual stresses and limited thicknesses. Thicker sol-gel PZT films require multiple coatings and high temperature annealing, which will cause serious stress issues. Moreover, since the piezoelectric properties of deposited thin films are largely dependent on the crystal orientation and substrate condition, proper buffer layers are required to prevent the material interdiffusion and oxidation and help to obtain good piezoelectric properties with lower residual stress.

The material properties of these commonly used piezoelectric thin films and the commercial ceramic PZT are summarized in Table 1. Since most of piezoelectric MEMS speakers work on the d_31_ mode of the piezoelectric layer, only the d_31_ piezoelectric constant is listed in the table for comparison. Among these materials, AlN thin films have the smallest piezoelectric constant, while PZT thin films exhibit the highest piezoelectric constant, which is about 10 to 20 times greater than that of ZnO thin films. However, the piezoelectric constant of PZT films also vary in a wide range, dependent on the film thickness, deposition, and poling conditions. In particular, the piezoelectric coefficient of the commercial ceramic PZT (e.g., PZT-5H) can reach 300 pm/V [71], which makes it a promising candidate for the construction of piezoelectric transducers.

#### 3.1.3. Approaches to Improve SPLs

Although a large number of piezoelectric MEMS speakers have been demonstrated based on various piezoelectric thin films with promising results, inadequate sound pressure level (SPL) outputs and non-flat frequency responses are common challenges of these devices. High SPLs of over 90 dB were achieved in a few piezoelectric MEMS speakers, but they were measured either in canals or ear simulators or at high-frequency resonances. Piezoelectric MEMS speakers with high SPLs (90 dB or above) over wide frequency ranges, especially in open air and low-frequency range, are needed for broader applications such as mobile phones, laptops, wearable electronics, and Internet of Things (IoT) devices. Therefore, several approaches have been proposed to improve the SPLs of piezoelectric MEMS speakers in terms of materials and fabrication processes and structure designs, which will be reviewed in the following.

Materials and Fabrication Processes

As discussed in Section 3.1.1, the commonly used piezoelectric thin films of ZnO and AlN deposited by sputtering or sol-gel methods suffer from large residual stresses and limited thickness. For sputtered or sol-gel PZT, their obtained piezoelectric constants are also not comparable with those of bulk piezoelectric crystals or ceramics. As illustrated in Table 1, the piezoelectric constant of ceramic PZT is over four times greater than that of sputtered or sol-gel PZT. Thus, ceramic PZT was gradually employed in fabricating the piezoelectric layer of MEMS speakers with particular fabrication process to thin down this material. In 2009, Kim et al. thinned ceramic PZT down to around 40 μm and fabricated piezoelectric MEMS speakers based on it, and they measured an SPL of 90 dB (±5 dB) in the audible frequency range under a 32-V_pp_ drive at 1 cm away from the MEMS speaker in an anechoic box [17]. The fabricated MEMS speaker also exhibited a total harmonic distortion (THD) of less than 15% from 400 Hz to 8 kHz. However, the acoustic diaphragm was as large as 20 mm × 18 mm.

**Figure 7:**
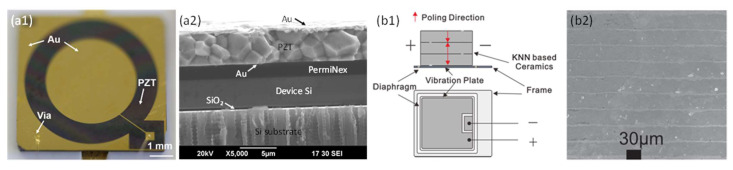
Piezoelectric MEMS speakers based on new materials: (**a1**) optical image and (**a2**) cross-section SEM image of a thin ceramic PZT-based MEMS speaker (Reproduced with permission from Elsevier [16]); (**b1**) schematic and (**b2**) cross-section SEM image of multilayer ceramics of a KNN ceramics-based MEMS speaker (Reproduced with permission from IOP [77]).

Since the resonant frequency of a diaphragm is affected by its area and thickness, scaling down the diaphragm size requires a thinner piezoelectric layer to maintain a proper resonant frequency. In 2020, Wang et al. presented a piezoelectric MEMS speaker based on thin ceramic PZT [16]. By using wafer bonding and chemical mechanical polishing techniques, ceramic PZT was thinned down to only 5 μm and applied to fabricate MEMS speakers. An optical image of the fabricated MEMS speaker and a cross-section SEM image of the device layers are shown in Figure 7a1,a2. Thin ceramic PZT not only exhibits much greater piezoelectric constants than sol-gel or sputtered PZT thin films but also has a wider range of thicknesses, thus allowing the scaling of diaphragms within size restrictions for different applications. With a 6 mm diameter diaphragm, the fabricated MEMS speaker achieved a maximum SPL of 119 dB measured at 1 cm under a 10-V_pp_ drive, as shown in Figure 9a [16].

Furthermore, lead-free piezoelectric ceramics with high piezoelectric constants have also been explored for fabricating piezoelectric MEMS speakers. For example, in 2014, Gao et al. fabricated piezoelectric MEMS speakers using potassium sodium niobate ((K,Na)NbO_3_, KNN)-based multilayer piezoelectric ceramics [77]. They employed a tape casting and cofiring process and used Ag–Pd alloys as an inner electrode. A schematic of the multilayer ceramics based piezoelectric MEMS speaker and a cross-section SEM image of the multilayer KNN-based ceramics are shown in Figure 7b1,b2, respectively. With a form factor of 23 × 27 × 0.6 mm^3^, using three layers of 30-μm-thick KNN-based ceramics, the fabricated MEMS speakers showed an average SPL of 87 dB from 1 kHz to 20 kHz measured at 3.16 cm under a 5-V_rms_ drive.

Structure Designs

As illustrated in Section 2.1, the output SPL of a MEMS speaker is directly determined by the frequency, area, and displacement of its diaphragm. Increasing the out-of-plane displacement of piezoelectric diaphragms is an effective approach to improve SPLs, especially at low frequency, as a much larger displacement is required at low frequency to achieve the same SPL at high frequency. Therefore, various designs of piezoelectric MEMS speakers have been proposed to improve their SPLs by changing the diaphragm structures, electrode configurations, or using an array form to enhance their acoustic performance.

Diaphragm Structures

**Figure 8:**
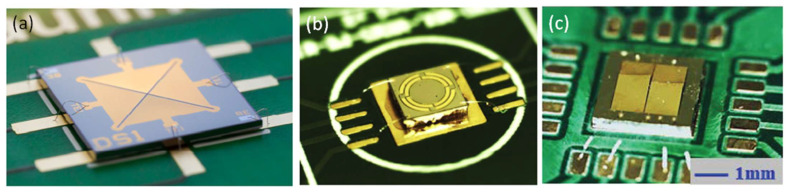
Optical images of piezoelectric MEMS speakers with novel structural designs. (**a**) A diaphragm with two open cuts (Reproduced with permission from IEEE [18]). (**b**) A diaphragm with suspension-spring actuators (Reproduced with permission from Elsevier [21]). (**c**) A diaphragm formed by four piezoelectric cantilevers with different dimensions (Reproduced with permission from IEEE [79]).

**Figure 9:**
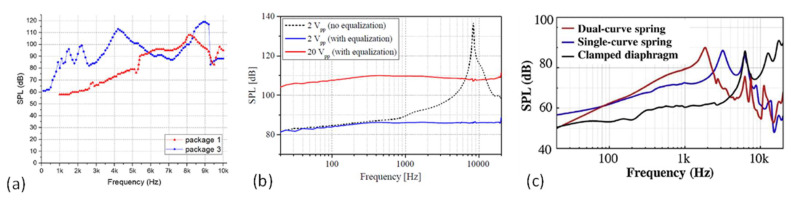
Measured frequency response of piezoelectric MEMS speakers with (**a**) a thin ceramic PZT-based diaphragm in free field at 1 cm (Reproduced with permission from Elsevier [16]), (**b**) a square diaphragm with open cuts in an ear simulator (Reproduced with permission from IEEE [18]), and (**c**) dual-curve spring actuators in a 3-cm-long tube (Reproduced with permission from Elsevier [21]).

In 2018, Stoppel et al. demonstrated a piezoelectric MEMS speaker based on a 2-μm-thick sputtered PZT with two open cuts on a square diaphragm (4 × 4 mm^2^) for in-ear applications, as shown in Figure 8a [18]. Without a closed diaphragm, four individual actuators are mechanically decoupled from each other and thus can achieve larger out-of-plane displacements. The measurement in an ear simulator showed a high SPL of above 81 dB from 20 Hz and above 100 dB from 4.7 kHz to 15.8 kHz under a 2-V_pp_ drive, as shown in Figure 9b. The measured THD was less than 2% at most frequencies, except for the subharmonics of the resonance frequency, where the THD was increased to 7%.

In 2020, Cheng et al. presented a piezoelectric MEMS speaker with enhanced SPL by designing suspension-spring actuators with a dual-electrode driving [21]. As shown in Figure 8b, the designed MEMS speaker consisted of a circular moveable diaphragm and four flexible spring actuators. Dual-curve spring actuators with dual-electrode driving were utilized to achieve larger displacements than single-curve spring actuators under the same form factor. Measurements in a 3-cm-long tube showed a maximum SPL of 90.1 dB at the resonance of 1.85 kHz under a 2-V_pp_ drive, which was 28 dB higher than the SPL of a fully clamped diaphragm speaker at the same frequency (Figure 9c). The measured THD of the dual-curve spring device was also lower than those of the clamped diaphragm devices, which was less than 2% at most frequencies and low than 8% at the resonant or harmonic frequencies.

In addition to employing unsealed vibration diaphragms with large displacements, Wang et al. proposed a rigid–flexible vibration coupling mechanism in 2021. By depositing a Parylene film on a pre-etched diaphragm, the fabricated MEMS speaker can maintain large displacements of the unsealed diaphragms without acoustic loss. Measurement in an ear simulator under a 2-V drive showed SPLs can exceed 59 dB from 250 Hz to 20 kHz, with the maximum value of 101.2 dB obtained at the resonance of 6.7 kHz [78].

To improve SPLs over a broad frequency range, in 2021, Wang et al. proposed a cantilever array design with an in-phase/out-of-phase hybrid driving method to realize a broadband piezoelectric MEMS speaker [79]. As shown in Figure 8c, the device consisted of four piezoelectric cantilevers with different dimensions, the four resonance frequencies of which contribute to the broadband performance of the MEMS speaker. In this device, in order to avoid the sound pressure cancellation due to the large phase shifts around the resonances of the cantilevers, a hybrid drive voltage with a combination of both in-phase and out-of-phase signals was applied to ensure that the cantilevers vibrate in the same direction. Measurements showed a broadband frequency response from 100 Hz to 10 kHz with an SPL of 70 dB or higher and a maximum SPL of 110 dB at 1.54 kHz in an ear simulator under a 2-V_pp_ drive.

Electrode Configurations

**Figure 10:**
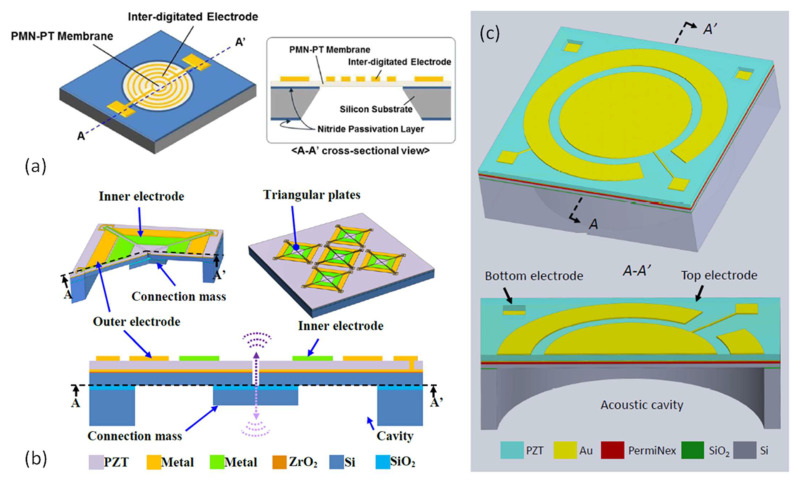
Schematics of piezoelectric MEMS speakers with special electrode configurations. (**a**) Circular inter-digitated electrode (Reproduced with permission from Springer [23]). (**b**) Triangular plates with dual electrode (Reproduced with permission from IEEE [56]). (**c**) Circular diaphragm with dual electrode (Reproduced with permission from IEEE [24]).

Efforts have also been devoted to improving the SPLs of MEMS speakers by the special design of electrode configurations. Electrode configurations on piezoelectric diaphragms are important as they largely determine the excitation mode, vibration displacement, and electromechanical coupling efficiency. As introduced in Section 2.1, most piezoelectric MEMS speakers work on the d_31_ flexural vibration mode of piezoelectric diaphragms with the electrical field applied in the thickness direction and the strain generated in the lateral directions. In addition to the d_31_ vibration mode, piezoelectric materials can also be excited in the d_33_ mode with the applied electrical field and the generated stain in the same direction, typically in the thickness direction. Typically, the magnitude of the d_33_ constant of a piezoelectric material is roughly two times larger than that of the d_31_ constant. Therefore, by proper electrode configurations, the d_33_ mode of piezoelectric diaphragms can be excited with larger out-of-plane displacements than the d_31_ mode. In 2015, Kim et al. presented a piezoelectric MEMS speaker based on the d_33_ mode PMN-PT single crystal diaphragm with a circular inter-digitated electrode (IDE) configuration and studied the effects of the patterned electrodes on the acoustic characteristics of the MEMS speaker [23]. A single crystal PMN-PT was thinned down to 10 μm to form an 8.5 mm diameter diaphragm by grinding, polishing, and inductively-coupled-plasma (ICP) etching, followed by metallization with circular IDE patterns on the top, as shown in Figure 10a. Measurements showed improved SPL with increasing area of the patterned IDE. With an 8 mm diameter IDE, the MEMS speaker showed an average SPL of above 70 dB from 1 kHz to 10 kHz and a maximum SPL of around 100 dB at 1 cm under a 5-V_rms_ drive.

In addition to the IDE configuration that can excite the piezoelectric d_33_ mode for SPL improvement, dual-electrode configuration has been investigated to improve the SPLs of piezoelectric MEMS speakers working on the d_31_ mode. In 2020, Tseng et al. presented a piezoelectric MEMS speaker with the SPL improved by dual-electrode driving [56]. The schematic of the designed MEMS speaker is shown in Figure 10b, where the square diaphragm consists of four triangular plates whose vibrations are synchronized by a connection mass. The low frequency response can be enhanced by reducing the size of the gaps between the triangular plates. Each triangular plate can be driven by an inner electrode and an outer electrode with a 180° phase difference to actuate the piston mode of the diaphragm to increase the SPL. Measurements showed a SPL enhancement of 9.5 dB under the dual-electrode driving in comparison with the single (inner or outer) electrode driving.

In addition to the 180° out-of-phase, other phase differences in dual-electrode driving and their influences on the SPL improvement of piezoelectric MEMS speakers have been studied. In 2021, Wang et al. presented a ceramic PZT-based piezoelectric MEMS speaker with the SPL improved by dual-electrode driving and studied the effects of the phase difference at different frequencies [24]. As shown in Figure 10c, the reported MEMS speaker consists of an inner circular electrode and an outer ring-shaped electrode. By applying sine waves on these two electrodes with a phase difference tuned from 0° to 360° in the experiments, the measurement results revealed that the SPL changed significantly with the phase difference and was frequency dependent, peaking at different phase differences for different frequencies. With the optimal phase differences, a 2–10 dB SPL improvement can be achieved in the frequency band spanning from 600 Hz to 10 kHz, compared with the single-electrode driving method.

Array Structures

**Figure 11:**
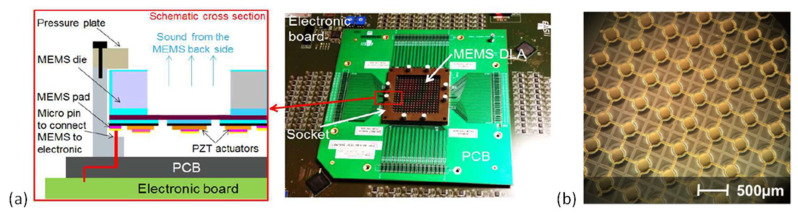
Schematic and optical images of piezoelectric MEMS speaker arrays: (**a**) a 256-speaker array packaged on an electronic board (Reproduced with permission from Elsevier [81]) and (**b**) part of a 1024-speaker array (Reproduced with permission from IEEE [82]).

Another approach to improve the SPLs of the piezoelectric MEMS speakers is using digital sound reconstruction or speaker arrays. Different from traditional sound generation techniques that rely on the vibration amplitudes and frequencies of a single or a few diaphragms to achieve high SPL at specific frequencies, digital sound reconstruction generates loud sound by adding the outputs of a large number of speaker pixels that can be excited individually by signals with different frequency compositions [80]. Typically, a speaker array containing 2^n^ speaker pixels is used in digital sound reconstruction, where *n* is the bit number, and each pixel contributes a small amount of sound pressure in the system. In 2015, Casset et al. implemented digital sound reconstruction with piezoelectric MEMS speaker arrays [81]. Figure 11a shows the fabricated speaker array packaged on an electronic board. With a chip size of 4 × 4 cm^2^, the speaker array contains 256 piezoelectric diaphragms based on a 2-μm sol-gel PZT film. The output SPL of the speaker array reached over 100 dB at 13 cm. In 2016, Arevalo et al. increased the bit number and presented a 10-bit (1024 elements) piezoelectric MEMS speaker array with a chip size of 2.3 × 2.3 cm^2^ [82]. An optical image of part of the speaker array is shown in Figure 11b. The characterization results demonstrated the potential of piezoelectric MEMS loudspeaker arrays for digital sound reconstruction, but more efforts are still needed to optimize the design for better acoustic performances.

#### 3.1.4. Summary of Piezoelectric MEMS Speakers

Piezoelectric MEMS speakers are reviewed above from piezoelectric materials, fabrication techniques, and approaches to improve SPLs. Table 2 summarizes the key results of these piezoelectric MEMS speakers. As shown in the table, sol-gel and sputtered PZT films are popular piezoelectric materials for fabricating piezoelectric MEMS speakers due to their higher piezoelectric constants than those of ZnO or AlN films. Piezoelectric MEMS speakers based on sol-gel or sputtered PZT films with thicknesses of 1–2 μm typically have diaphragm sizes of no more than 4 mm and can generate high SPLs over 90 dB in tubes or ear simulators for in-ear applications. With optimized structure designs, their SPLs can be significantly improved to reach maximum values over 110 dB under small driving voltages. Moreover, piezoelectric MEMS speakers based on ceramic PZT or single-crystal PMN-PT can generate high SPLs in open air, which have potential applications in consumer electronics such as cell phones or laptops. Bulk ceramic PZT or PMN-PT with superior piezoelectric properties can be thinned down to 5–40 μm for fabricating piezoelectric MEMS speakers, which enables larger diaphragm designs ranging from 6 mm to 2 cm and high SPLs of over 100 dB at 1 cm in open air.

### 3.2. Electrodynamic MEMS Speakers

Electrodynamic MEMS speakers have been developed based on electromagnetic actuation, which is the most widely used actuation mechanism in classical speakers. Electrodynamic MEMS speakers have advantages of high power density, low driving voltage, and linear responses. Efforts have been devoted to the development of electrodynamic MEMS speakers with integrated magnetic materials and small form factors at low cost, while improving their sound performances. However, the full integration of magnetic materials to realize electrodynamic MEMS speakers is still challenging.

**Figure 12:**
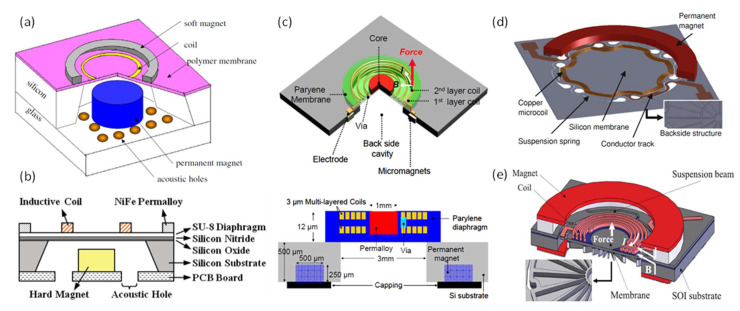
Electrodynamic MEMS speakers: (**a**) typical structure of an electrodynamic MEMS speaker (Reproduced with permission from IOP [5]), (**b**) cross-sectional view of a low-power electrodynamic MEMS speaker with Cu–Ni nanocomposite coil synthesized (Reproduced with permission from Journal of IEEE [83]), (**c**) schematic (top) and cross-sectional view (bottom) of a fully integrated electrodynamic MEMS speaker (Reproduced with permission from IEEE [15]), (**d**) schematic of an electrodynamic MEMS speaker with a rigid silicon diaphragm (Reproduced with permission from Springer [84]), (**e**) schematic of an electrodynamic MEMS speaker showing a rigid silicon diaphragm and the optimized configuration of coil and two face-to-face magnets (Reproduced with permission from IEEE [2]).

**Figure 13:**
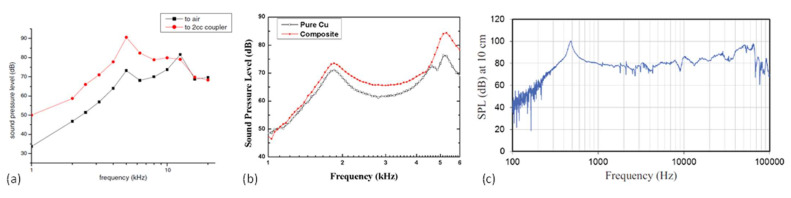
Typical frequency responses of electrodynamic MEMS speakers based on (**a**) a polymer diaphragm and a Cu coil and measured at 2 cm in air and in a 2-cc coupler (Reproduced with permission from IOP [5]), (**b**) pure Cu and Cu–Ni composite coils and measured in a 2-cc coupler (Reproduced with permission from IEEE [83]), (**c**) a rigid silicon diaphragm and measured at 10 cm in air (Reproduced with permission from IEEE [2]).

In 2004, Cheng et al. presented an electrodynamic MEMS speaker for hearing instruments. The device was fabricated with a low temperature process using an electroplated Ni/Fe soft magnet, which was suitable for post-CMOS processing and potential integration with electronic circuits [5]. A schematic of the designed MEMS speaker is shown in Figure 12a, which has a chip size of 5 mm × 5 mm and consists of a micromachined polymer diaphragm on a silicon wafer, a single-curve Cu coil, an electroplated Ni/Fe soft magnet, and a permanent magnet mounted on the backside. The frequency responses of the fabricated device are measured in air and in a 2-cc coupler with results shown in Figure 13a. At a low driving voltage of 1.5 V, the MEMS speaker generated a maximum SPL of 93 dB at 5 kHz in a 2-cc volume. This work provided a concept and process for micromachining electrodynamic MEMS speakers. Following that, several electrodynamic MEMS speakers have been reported for lower power consumption, high-level integration process, and improved SPL and sound quality.

In 2009, Chen et al. presented an electrodynamic MEMS speaker with improved power efficiency through incorporating Ni nano-composites into Cu to make the voice coil [83]. A cross-sectional view of the MEMS speaker structure is illustrated in Figure 12b, where the coil is made of a Cu–Ni composite by mixing Ni nano-powders with alkaline non-cyanide-based copper-plating solution in a colloidal bath. The frequency responses of the fabricated MEMS speakers driven by the Cu–Ni nanocomposite and pure Cu coils are measured and compared, as shown in Figure 13b. The experimental results showed that the MEMS speaker with a Cu–Ni composite coil can averagely provide about 40% power savings than the one with a Cu coil for the same SPL output at 70 dB.

As shown in Figure 12a,b, most electrodynamic MEMS speakers require the assembly of a bulky permanent magnet, which will not only increase the overall footprint of the device but also add challenge to the batch fabrication process and precise alignment of the magnet to the diaphragm coil. In order to address this issue, in 2009, Je et al. presented a fully-integrated electrodynamic MEMS speaker with an IC process-compatible micromachined permanent magnets for hearing aid applications [15]. A schematic and cross-sectional view of the presented MEMS speaker is shown in Figure 12c, where a Parylene diaphragm containing embedded multi-turn coils and a soft magnet core is suspended over an acoustic cavity. A rare earth Nd–Fe–B magnetic powder was mixed into a wax binder and dispensed into pre-etched trenches to form the permanent ring micromagnet. The fabricated MEMS speaker produced a 0.64 μm peak displacement at 1 kHz with a 46-mW power consumption. Referring to Figure 2, the achieved displacement is too small to generate sufficient SPLs by a diaphragm with a diameter of 3 mm. Although this work demonstrated the feasibility of fabricating fully integrated electrodynamic MEMS speakers, further design optimization is required to improve the displacement and acoustic performance.

Most MEMS speakers use clamped polymer diaphragms, such as polyimide, Parylene, and SU-8, for flexural vibration and sound generation, whose small mass is in favor of power efficiency and large deflection. However, the flexible nature of polymer diaphragms will lead to dynamic deformations and numerous structural modes within the audio frequency band, thus inducing sound distortion and non-flat frequency response. From 2012 to 2013, to improve the sound performance of electrodynamic MEMS speakers, Shahosseini et al. proposed novel electrodynamic MEMS speakers based on rigid silicon diaphragms and optimized structural designs [2,6,37,84]. The rigid silicon diaphragms were designed with radial ribbed structures for increased stiffnesses and reduced masses, thus enhancing the piston mode vibration for the sound generation and shifting other modes out of the audio frequency band. Figure 12d shows the structure of such an electrodynamic MEMS speaker, where the silicon diaphragm was connected to the substrate by a set of flexible springs to provide out-of-plane displacements [84]. A 14-turn Cu coil was shaped in a special geometry to prevent the damage near the springs’ clamp areas, and it was located as close as possible to the permanent magnet to maximize the electromagnetic force. The same research group also investigated the distribution of the magnetic flux density under different configurations of the permanent magnets. In 2013, another electrodynamic MEMS speaker with an optimized microcoil configuration and two face-to-face magnets has been developed [2], as shown in Figure 12e. The fabricated MEMS speaker had a circular diaphragm with a diameter of 15 mm and generated a SPL of around 80 dB at 10 cm starting from 300 Hz to over 20 kHz, as shown in Figure 13c.

Table 3 summarizes the key results of these electrodynamic MEMS speakers. As shown in the table, electrodynamic MEMS speakers based on polymer diaphragms typically have small size and low power consumption but limited SPLs. Their maximum SPLs are around 100 dB or less measured in 2-cc couplers or ear simulators. By contrast, electrodynamic MEMS speakers with rigid silicon diaphragms can generate loud sound in open air at large distance but suffer from large diaphragm size and high power consumption.

### 3.3. Electrostatic MEMS Speakers

MEMS speakers based on electrostatic actuation have also been proposed, which typically consist of parallel or lateral plate actuators. The advantages of such speakers include easy fabrication, high electromechanical efficiency, and relatively flat frequency response. In this section, the recent designs of electrostatic MEMS speakers based on different diaphragm materials will be introduced first. Then, the approaches to improve SPLs of electrostatic MEMS speakers while balancing the design constraints will be reviewed in detail.

#### 3.3.1. Devices with Different Diaphragm Materials

**Figure 14:**
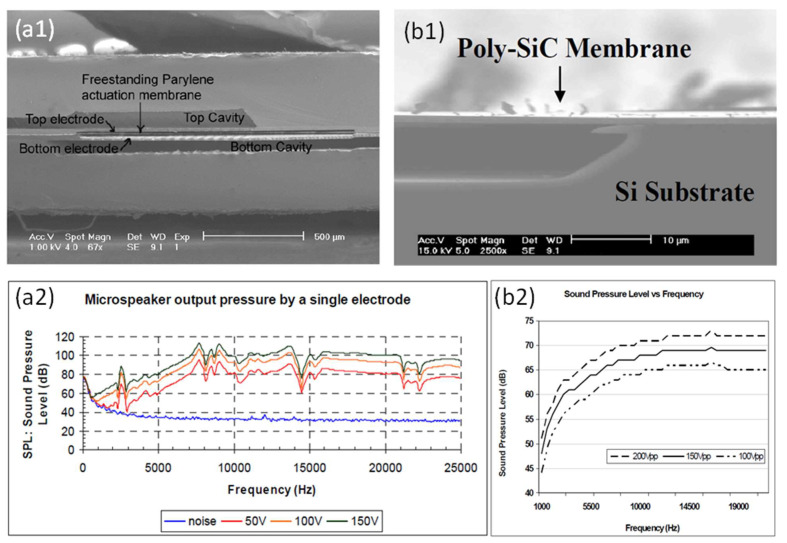
Electrostatic MEMS speakers based on different diaphragm materials: (**a1**) SEM image and (**a2**) measured frequency response of a bi-directional MEMS speaker with a Parylene diaphragm (Reproduced with permission from IEEE [88]). (**b1**) SEM image and (**b2**) measured frequency response of a touch-mode MEMS speaker with a poly-SiC diaphragm (Reproduced with permission from IEEE [8]).

Electrostatic MEMS speakers have been demonstrated based on different diaphragm materials [8,39,87,88]. In 2005, Kim et al. reported an electrostatic MEMS speaker based on a Parylene thin diaphragm. As the cross-sectional SEM image shown in Figure 14a1, the speaker contains two separated chambers on the top and bottom, respectively, which enables bi-directional actuation by electrostatic forces [88]. Figure 14a2 shows the measured frequency response of the speaker. With a diaphragm size of 2 × 2 mm^2^, the fabricated device generated high SPLs of 113.4 dB at 7.68 kHz and 98.8 dB at 13.81 kHz, which were measured at a distance of 1 cm under a driving voltage of 150 V. In 2007, Roberts et al. presented an electrostatically driven touch-mode MEMS speaker based on poly-SiC diaphragms with a diameter of 800 μm, which was robust and operable in harsh environments [8]. Figure 14b1 shows the SEM image of the suspended poly-SiC diaphragm of the fabricated device. At a distance of 1 cm, a maximum SPL of 73 dB was obtained at 16.59 kHz under a driving voltage of 200 V (Figure 14b2). Another material, graphene, has also been explored for fabricating high-quality broad-band audio speakers due to its extremely low mass density and high mechanical strength. In 2013, Zhou et al. presented a miniaturized electrostatic speaker based on a 30 nm thin graphene diaphragm and demonstrated a broad frequency response from 20 Hz to 20 kHz with the performance matching or surpassing a commercial magnetic coil speaker [39].

#### 3.3.2. Approaches to Improve SPLs

Most electrostatic MEMS speakers are based on the conventional parallel plate structures and have low SPLs due to the small deflections of their diaphragms, which is a direct result of the balance between the electrostatic force and the mechanical restoring force. In order to overcome the limitation of low SPLs, large electrostatic forces need to be generated. As introduced in Section 2.2 and shown in Equation (7), large electrostatic forces require high driving voltages and small separation gaps. However, the small separation gap will limit the deflection range of the diaphragm and generate large squeeze film air damping [89]. Moreover, the driving voltage must be reasonably lower than the pull-in voltage of the parallel plates to ensure a good reliability. Therefore, tradeoffs have to be made among the electrostatic force, the separation gap between the parallel plates, and the driving voltage to increase the SPLs of electrostatic MEMS speakers.

**Figure 15:**
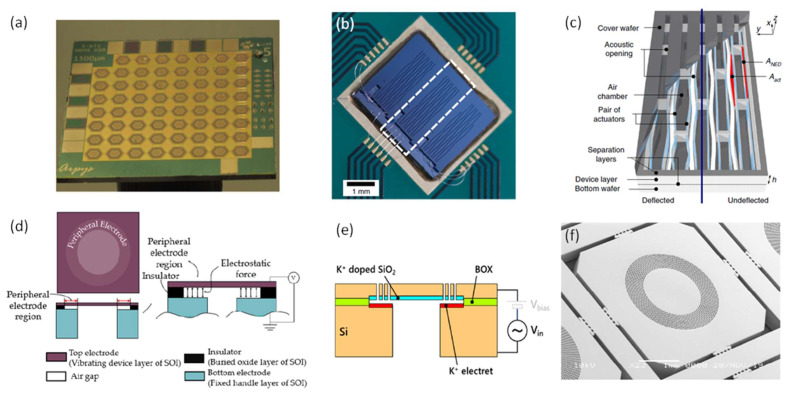
Electrostatic MEMS speakers with special designs: (**a**) optical image of a MEMS speaker array (Reproduced with permission from IEEE [91]), (**b**) schematic and (**c**) optical image of a MEMS speaker with in-plane bending electrostatic actuators working in air chambers (Reproduced with permission from Nature Portfolio [9]), (**d**) schematic of a peripheral electrode configuration (Reproduced with permission from IEEE [38]), (**e**) schematic and (**f**) the corresponding SEM image of an electret-augmented MEMS speaker (Reproduced with permission from MDPI AG [92]).

To generate considerable SPLs and balance the above-mentioned constraints, several approaches in terms of device structure and driving voltage have been applied in the development of electrostatic MEMS speakers [9,38,90,91,92]. One approach to improve SPL is to use multiple speakers, i.e., array structures. In 2016, Arevalo et al. presented an electrostatic MEMS speaker array for digital sound generation, where each of the individual MEMS speakers had a hexagonal diaphragm connected to an outer hexagonal ring by tethers (Figure 15a) [91]. This work demonstrated the feasibility of generating sounds with electrostatic MEMS speaker arrays but lacked acoustical characterization results.

Different from conventional MEMS speakers that work on the out-of-plane deflection of a diaphragm, Kaiser et al. proposed a novel structure design in 2019, which consisted of in-plane bending electrostatic actuators working in air chambers based on the so-called nanoscopic electrostatic drive (NED) technology, as shown in Figure 15b [9,48]. Utilizing the curvy geometric shape of the moving beams, electrostatic forces are translated into lateral forces and cause the bending of the beams. Therefore, high SPLs can be reached by the large deflection of the beams in the air chambers and a large number of beams in one chip, without the limitation of small separation gaps between electrodes [93]. This novel structure utilized the chip’s bulk volume rather than the surface to generate sound pressures. Figure 15c shows an optical image of such a fabricated electrostatic MEMS speaker with in-plane actuators. The acoustic measurement in an ear simulator showed a SPL of 69 dB at 500 Hz with a THD of 4.4%. The maximum SPL reached 104 dB at 11.4 kHz.

In 2020, Garud et al. designed and fabricated a MEMS speaker with peripheral electrostatic actuation [38]. Figure 15d shows the schematic of the designed electrostatic MEMS speaker, where the clamped circular diaphragm has a peripheral electrode configuration that can mitigate the squeeze film damping effect and increase the pull-in voltage. The simulation results showed that as the peripheral electrode width was reduced from 100% (full electrode coverage) to 10%, the pull-in voltage and the vibration amplitude of the diaphragm could be increased by a factor up to 40 and 80, respectively.

To reduce or eliminate the DC bias of electrostatic MEMS speakers, electrets embedded with quasi-permanent electrical charges have been integrated within the electrode structures. In 2020, Sano et al. presented an electret-augmented electrostatic MEMS speaker and demonstrated its sound generation under low driving voltages [92]. The schematic and an SEM image of the fabricated MEMS speaker are shown in Figure 15e,f, respectively. By integrating the electrets into the MEMS speaker, the built-in electrical potential is equivalent to an external DC bias, thus resulting in an increased displacement or a reduced bias voltage. The characterization result showed that a −10 V electret-augmented electrostatic MEMS speaker reached a maximum SPL of 50 dB at 1.5 cm under a 5-V_pp_ AC driving voltage.

Table 4 summarizes the representative electrostatic MEMS speakers reported in the literature. It can be seen that electrostatic MEMS speakers typically require high driving voltage and large DC bias to generate considerable diaphragm deflection. Most of electrostatic MEMS speakers have small separation gaps (1–8 μm) and limited sound pressure output. High SPLs are generally obtained only at the high-frequency range.

### 3.4. Thermoacoustic MEMS Speakers

In addition to the above reviewed three major types of transduction mechanisms, thermoacoustic transduction also has potential to be applied for making MEMS speakers. Several thermoacoustic loudspeakers have been developed based on carbon nanotube or graphene with research efforts focused on increasing the sound pressure output and reducing the power consumption [13,14,42,94,95].

**Figure 16:**
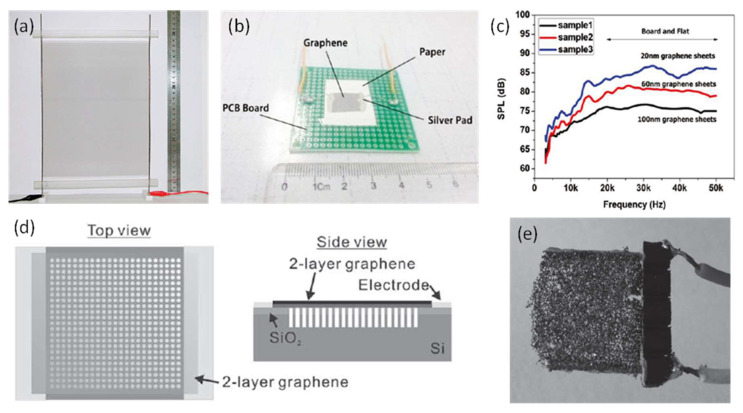
Thermoacoustic speakers: (**a**) photograph of a A4 paper size carbon nanotube thin film thermoacoustic speaker (Reproduced with permission from ACS [42]), (**b**) photograph and (**c**) measured SPL of a graphene-on-paper speaker (Reproduced with permission from ACS [94]), (**d**) schematic of a graphene speaker on a patterned substrate (Reproduced with permission from Wiley [14]), (**e**) photograph of a graphene foam speaker (Reproduced with permission from Wiley [13]).

In 2008, Xiao et al. found that thin carbon nanotube films emitted sound when a current in audio frequency was applied, which could be attributed to the thermoacoustic effect [42]. Based on this finding, they successfully fabricated thermoacoustic speakers with A4 paper sizes and cylindrical shapes (9 cm diameter and 8.5 cm height) based on one-layer or four-layer carbon nanotube thin films, which could generate over 70 dB SPLs at 5 cm starting from 1 kHz, with an input power of 3 Watts. Figure 16a shows the photograph of a fabricated thermoacoustic speaker with an A4 paper size. This work demonstrated the feasibility of developing thermoacoustic speakers using carbon nanotube films. However, it required a large device size and a high power consumption to generate high SPLs.

In 2011, Tian et al. observed thermoacoustic effect on graphene and demonstrated graphene-on-paper speakers [94]. As shown in Figure 16b, the fabricated thermoacoustic speaker had a 1 cm × 1 cm graphene sheet, which was placed on a piece of paper and connected to a printed circuit board (PCB) using silver ink. Graphene sheets with thicknesses of 20 nm, 60 nm, and 100 nm were used to fabricate speakers. Figure 16c shows the SPL curves with the input power density normalized to 1 W/cm^2^, which indicated that thinner graphene sheets produced higher SPLs and the SPL of 20 nm graphene sheets reached 85 dB at 5 cm with the frequency increased to over 15 kHz.

The sound performance of thermoacoustic speakers has been further studied and optimized in terms of substrate material and structure design. For example, in 2012, Suk et al. studied thermoacoustic sound generation with graphene on different substrates, including glass, polyethylene terephthalate (PET), and polydimethylsiloxane (PDMS) [14]. The substrate effect was also investigated by transferring graphene onto patterned substrates with different porosities, as shown in Figure 16d. The experiments revealed that graphene on the substrates with lower thermal effusivity and higher porosity exhibited better sound performances. In 2015, Fei et al. presented a low-voltage driven thermoacoustic speaker based on graphene foam synthesized by the nickel-template chemical vapor deposition (CVD) method [13]. A photograph of the fabricated free-standing graphene foam speaker is shown in Figure 16e. Benefited from high thermal conductivity and low in-plane resistance of the 3D graphene foam, the speaker generated a SPL of around 50 dB at 3 cm and 10 kHz with a power consumption of only 0.1 W.

In summary, thermoacoustic speakers made of carbon nanotubes or graphene films have advantages of simple structure, light weight, and easy fabrication. The transparent and stretchable nature of carbon nanotube or graphene films also makes it possible to fabricate them into any shape and size, freestanding or on any insulating surfaces, showing great potentials to be applied for developing thermoacoustic MEMS speakers. However, current thermoacoustic speakers require large size (1–4 cm) and high power consumption (0.1–3 W) to generate adequate sound pressure output.

## 4. Comparison of Different MEMS Speakers

As reviewed in Section 2 and Section 3, MEMS speakers have been demonstrated based on piezoelectric, electrodynamic, electrostatic, and thermoacoustic transduction mechanisms, showing great potentials for various applications including hearing instruments and portable electronic devices. Among them, piezoelectric MEMS speakers and electrodynamic MEMS speakers are the dominant types of MEMS speakers, which have been extensively studied and reported in a vast amount of the literature. Piezoelectric MEMS speakers have advantages of relatively large driving force and high sound pressure output over other MEMS speakers. High SPLs of over 90 dB have been achieved by several piezoelectric MEMS speakers either in ear simulators or in open air. Piezoelectric thin films including ZnO, AlN, PZT, and PMN-PT have been fabricated either by deposition or thinning down bulk materials and applied for fabricating piezoelectric MEMS speakers. However, most current piezoelectric MEMS speakers suffer from non-flat frequency responses due to the resonance behavior of diaphragms. The nonlinearity and hysteresis of piezoelectric materials are also drawbacks of piezoelectric MEMS speakers.

By contrast, electrodynamic MEMS speakers with quasi-linear behaviors are favorable for high-fidelity sound reconstruction. Low power consumption and large mechanical displacements are also advantages of electrodynamic MEMS speakers. Several electrodynamic MEMS speakers have been developed based on polymer diaphragms or rigid silicon diaphragms, with SPLs of 60–100 dB obtained in 2-cc couplers for in-ear applications. However, the requirement of permanent magnets for electrodynamic MEMS speakers not only increases the overall size of devices but also makes the full integration complicated and challenging.

In comparison, electrostatic MEMS speakers do not require complicated fabrication processes but suffer from small displacements, very high driving voltages, and pull-in limitations. Several approaches, such as nanoscopic electrostatic drive (NED) technology, have been proposed to balance the driving voltage, pull-in limitation, and displacement of the diaphragm. Improved SPLs and low THDs have been obtained on these electrostatic MEMS speakers.

Compared with piezoelectric, electrodynamic, and electrostatic MEMS speakers, thermoacoustic MEMS speakers are special acoustic devices that do not rely on mechanical vibration of diaphragms to generate sounds. Therefore, there are no resonant peaks in the frequency response of thermoacoustic MEMS speakers. High transparency, high stretchability, and easy fabrication into any sizes and shapes are the advantages of thermoacoustic MEMS speakers. However, current thermoacoustic speakers all require much larger sizes to achieve comparable SPLs of piezoelectric or electrodynamic MEMS speakers. Large power consumption is another concern of thermoacoustic MEMS speakers.

In common, all these MEMS speakers are required to improve their SPLs at specific frequencies to satisfy a wider range of applications. Approaches have been proposed and demonstrated on these MEMS speakers with improved SPLs, including applying new materials and fabrication processes, designing novel structures and special electrode configurations, and using large speaker arrays.

## 5. Summary and Outlook

In summary, MEMS speakers have been reviewed in terms of the theory, modeling, transduction mechanisms, and development history in this article. Four types of MEMS speakers, working on piezoelectric, electrodynamic, and electrostatic actuation and the thermoacoustic effect have been introduced; their respective development milestones, performances, advantages, and limitations are also discussed. Approaches to improve the SPLs of MEMS speakers including special structures, new materials, electrode configurations, and speaker arrays are highlighted and discussed, especially for piezoelectric MEMS speakers.

In the future, the SPLs of all types of MEMS speakers will continue to be improved by the incorporation of new materials, novel fabrication techniques, and optimized device and enclosure designs, as well as with deeper understandings of their modeling. In addition to SPLs, fabrication challenges, frequency response, sound quality, and power consumption will also be taken into account. Particularly, piezoelectric MEMS speakers will be extensively investigated to obtain flat frequency responses. Electrodynamic MEMS speakers will be further studied with electroacoustic efficiency improved and permanent magnets fully integrated in batch processes. Electrostatic MEMS speakers, with efforts in reducing driving voltages, and high-level integration with electronic circuits, may find broader applications, especially in digital sound reconstruction. Finally, thermoacoustic MEMS speakers will continue to be explored with efforts to reduce the device size and power consumption. Thereby, MEMS speakers are expected to become a promising candidate not only in the in-ear applications but also in a wide range of consumer electronics.

## Figures and Tables

**Table 1:** Material properties of commonly used piezoelectric thin films and the commercial ceramic PZT [21,71,72,73,74,75,76].

Property	ZnO	AlN	Sol-Gel PZT	Sputtered PZT	Ceramic PZT-5H
Density (kg/m^3^)	5700	3260	7700	7700	7800
Young’s modulus (GPa)	98.6	283	96	96	50
Dielectric constant	8.8	8.5–10.7	650–1470	400–980	3400
Piezoelectric constant d_31_ (pm/V)	3.9–5.5	2–2.6	23–76	45–102	270–300

**Table 2:** Key results of different piezoelectric MEMS speakers.

	Ref.	Piezoelectric Layer	Diaphragm Size	1st Resonant Frequency	Maximum SPL	Driving Voltage	Note
In-coupler measurement	[58]	0.5 μm ZnO	2 mm length (square)	890 Hz	100 dB at 4.8 kHz	12 V_pp_	Measured into a 2 cm^3^ coupler
	[21]	1 μm sputtered PZT	1.13 mm diameter (central part)	1.85 kHz	90.1 dB at 1.85 kHz	2 V_pp_	Measured in a 3-cm-long tube
	[78]	1 μm sputtered PZT	2 mm side length (hexagon)	6.7 kHz	101.2 dB at 6.7 kHz	2V (unspecified)	Measured in an ear simulator
	[79]	2 μm sputtered PZT	4 mm^2^ (rectangle)	1.54 kHz	110 dB at 1.54 kHz	2 V_pp_	Measured in an ear simulator
	[56]	2 μm sputtered PZT	3.24 mm^2^ (four triangles)	~6 kHz	118.1 dB at 11.9 kHz	2 V_pp_	5-speaker array, measured in an ear simulator
	[18]	2 μm sputtered PZT	4 mm length (square)	8.3 kHz	138 dB at 8.3 kHz	2 V_pp_	Measured in an ear simulator
Free-field measurement	[64]	0.5 μm ZnO	3 mm length (square)	7.3 kHz	83.1 dB at 13.3 kHz	30 V_pp_	Measured at 1 cm
	[10]	0.5 μm ZnO	5 mm length (square)	2.92 kHz	92.4 dB at 2.92 kHz	6 V_pp_	Measured at 2 mm
	[36]	0.5 μm AlN	4 mm length (square)	−	100 dB at 10 kHz	20 V_pp_	Measured at 3 mm
	[35]	0.5 μm AlN	−	−	104 dB at 3 kHz	20 V_pp_	Device in a 4 cm^3^ package, measured at 1 cm
	[11]	0.7 μm sol-gel PZT	2 mm diameter	−	90 dB at 10 kHz	13 V (unspecified)	Measured at 1 cm
	[81]	2 μm sol-gel PZT	2.6 mm diameter	18 kHz	~110 dB	8 V (unspecified)	256-speaker array, measured at 13 cm
	[16]	5 μm ceramic PZT	6 mm diameter	4.3 kHz	119 dB at 9 kHz	10 V_pp_	Measured at 1 cm
	[17]	40 μm ceramic PZT	18 mm × 20 mm	0.49 kHz	~106 dB at 5.5 kHz	32 V_pp_	Measured at 1 cm
	[23]	10 μm PMN-PT	8.5 mm diameter	1.4–1.84 kHz	~100 dB at 6.5 kHz	10$\sqrt{2}$ V_pp_	Measured at 1 cm

**Table 3:** Key results of different electrodynamic MEMS speakers.

Ref	Diaphragm Material	Diaphragm Size	Maximum SPL	Power Consumption	Note
[5]	Polyimide	3.5 mm diameter	93 dB at 5 kHz	320 mW	Measured in a 2 cm^3^ volume
[7]	Polyimide	3 mm diameter	106 dB at 1 kHz	0.13 mW	Calculated based on the displacement
[85]	Polyimide	2.5 mm diameter	90 dB at 1,5,10 kHz	−	Measured in a sealed 1500 mm^3^ silicone tube
[83]	SU-8	-	Around 85 dB at 5.2 kHz	−	Measured in a 2 cm^3^ volume
[86]	PDMS	3.5 mm diameter	106 dB at 1 kHz	1.76 mW	Measured in a 2 cm^3^ volume
[2]	Silicon	15 mm diameter	80 dB at 0. 33 kHz	0.5 W	Measured at 10 cm

**Table 4:** Key results of different electrostatic MEMS speakers.

Ref	Diaphragm Size	Electrode Separation	Maximum SPL	Driving Voltage	Note
[92]	2 mm diameter	2 μm, peripheral electrode	50 dB at around 35 kHz	AC 5 V_pp_	Measured at 1.5 cm
[8]	0.8 mm diameter	8 μm, touch mode	73 dB at 16.59 kHz	AC 200 V_pp_	Measured at 1 cm
[38]	3.1 mm diameter	1 μm, peripheral electrode	75–78 dB at above 10 kHz	DC 30 V + AC 30 V	Measured at 1 cm
[88]	2 mm length (square)	7.5 μm	113.4 dB at 7.68 kHz	AC 150 V	Measured at 1 cm
[9]	-	-	104 dB at 11.4 kHz	DC 40 V + AC 10 V_pp_	Measured in an ear simulator

## Data Availability

Not applicable.
